# p62 mRNA suppresses NLRP1 expression in cutaneous SCC cells through miR-34a-5p

**DOI:** 10.1038/s41419-025-07785-9

**Published:** 2025-07-01

**Authors:** Paulina Hennig, Patrick Turko, Michela Di Filippo, Mitchell P. Levesque, Thomas Kündig, Hans-Dietmar Beer

**Affiliations:** 1 Department of Dermatology, University Hospital Zurich, CH-8952 Schlieren, Switzerland; 2 Faculty of Medicine, University of Zurich, CH-8032 Zurich, Switzerland

**Keywords:** Translational research, Cancer

## Abstract

The inflammasome sensor NLRP1 is mainly expressed by epithelial cells including keratinocytes of human skin. Germline gain-of-function mutations in *NLRP1* cause inflammatory skin syndromes and predispose patients to the development of cutaneous squamous cell carcinomas (cSCCs), a major type of skin cancer originating from keratinocytes. However, expression of NLRP1 is strongly reduced in cSCCs suggesting a complex role of the NLRP1 inflammasome in the development of this type of skin cancer. Suppression of NLRP1 expression in SCC cells is partially caused by an increase in p62 (*SQSTM1*), a cargo receptor for autophagy-dependent protein degradation. p62 is upregulated in numerous types of cancer and plays key roles in tumor development by activating different pathways. Here, we characterized the molecular mechanisms underlying suppression of NLRP1 expression by p62 in cSCCs. In SCC cells, NLRP1 activation is rescued by a knockdown or knockout of p62 mRNA and, consequently, protein expression, rather than by a knockout of p62 protein expression only. As these experiments suggest a regulation of NLRP1 by the p62 mRNA, we characterized p62 mRNA-regulated gene expression in SCC cells through RNA sequencing. In addition to mRNAs, we identified several differentially regulated microRNAs (miRs), including miR-34a-5p. These short non-coding RNAs regulate the stability or translation of mRNAs in a dynamic manner and a single miR can target multiple mRNAs. miR-34a-5p is an established tumor suppressor in different types of cancer and its expression is also downregulated in cSCCs. Although miR-34a-5p seems to bind neither p62 nor NLRP1 mRNA directly, it increases NLRP1 expression, most likely through an indirect and complex mechanism, which occurs at the RNA level. In summary, our findings revealed a novel pathway regulating suppression of the inflammasome sensor NLRP1 in SCC cells by p62, which occurs at the mRNA level and is mediated by miRs, including the tumor suppressive miR-34a-5p. Therefore, a pharmacological increase in miR-34a expression represents a treatment option for cSCC patients that allows not only to target know proteins regulated by miR-34a but also a reconstitution of NLRP1 expression.

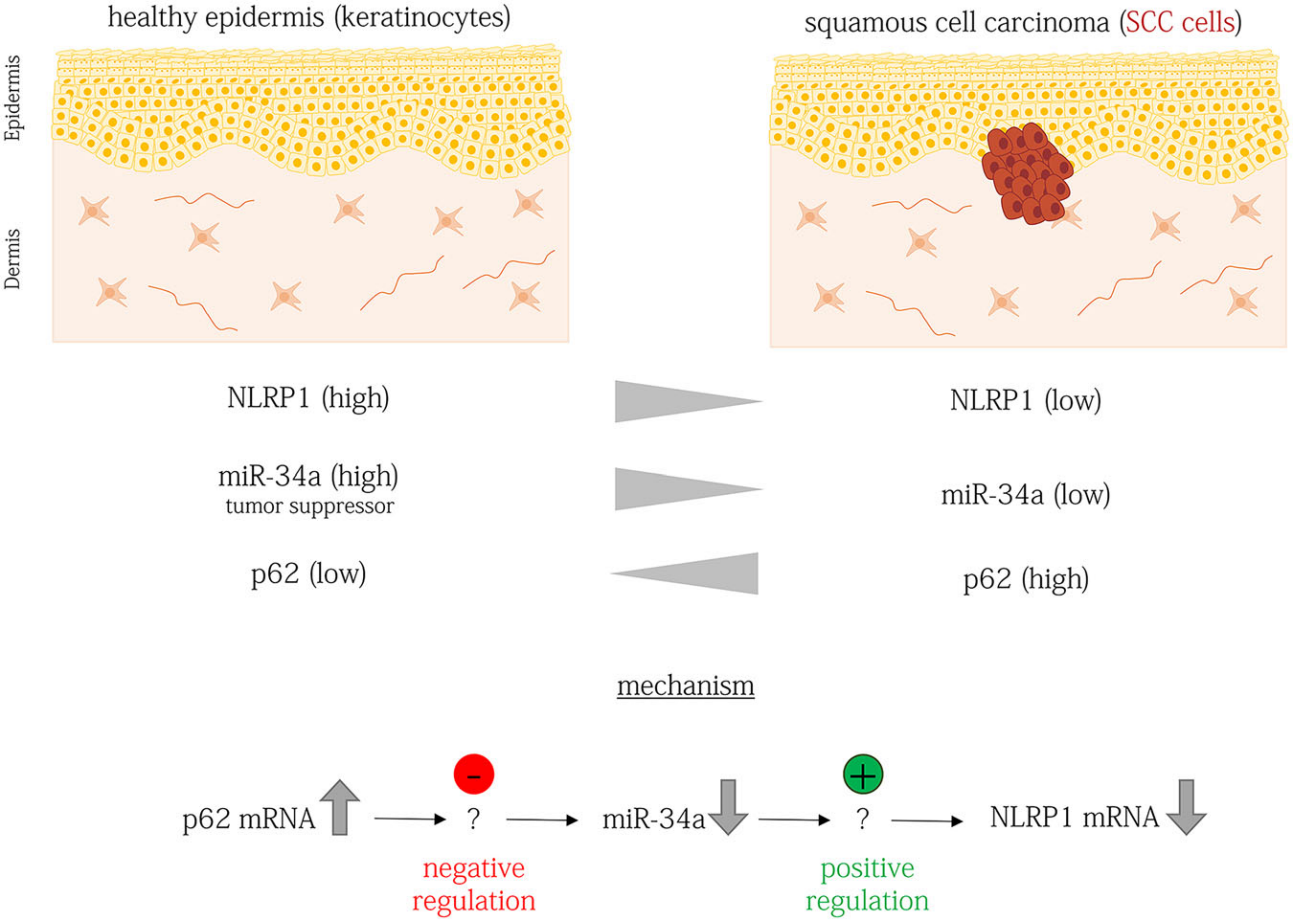

## Introduction

The epidermis is our body’s first line of defense against numerous physical, chemical and biological threats [1]. Through its permanent renewal and its stratified structure, consisting of several layers of keratinocytes in distinct differentiation stages, it represents an efficient outer barrier. Nevertheless, keratinocyte-derived cancer, comprising mainly of basal cell carcinoma (BSC) and cutaneous squamous cell carcinoma (cSCC), is the most prevalent type of cancer worldwide and cSCC after melanoma the second most mortal type of skin cancer [2]. UVB is the main risk factor for skin cancer, as it is absorbed by the DNA of epidermal cells leading to damage and mutations [3]. Furthermore, UVB is absorbed by RNA and thereby activates the ribotoxic stress response, which is believed to underlie the induction of sunburn [4]. However, UVB causes also immunosuppression through mechanisms which remain partially understood, contributing to photocarcinogenesis [5]. An important role of immunosurveillance in cSCC development is reflected by the fact that the incidence of cSCCs is strongly increased in immune-suppressed patients [6].

Nod-like receptor family pyrin domain containing 1 (NLRP1) is a critical mediator of inflammation in different epithelia, including the epidermis, and is highly expressed by human keratinocytes [7]. Germline gain-of-function mutations of *NLRP1* cause rare inflammatory skin syndromes and cSCC predisposition, whereas single nucleotide polymorphisms are associated with several common inflammatory skin diseases [8]. NLRP1 is a sensor protein of inflammasomes, which comprise a family of protein complexes activated upon sensing of different stress factors [9]. Most inflammasomes, such as the NLRP3 or AIM2 inflammasome, are expressed by immune cells [9]. These protein complexes are required for immunity, but their aberrant and chronic activation underlies the pathophysiology of numerous inflammatory diseases, such as diabetes or cardiovascular diseases, and cancer [10, 11]. Inflammasome activation induces activation of the protease caspase-1, which subsequently activates the pro-inflammatory cytokines interleukin (IL)-1β and IL-18 and gasdermin D (GSDMD). Once activated, GSDMD forms pores in the cell membrane allowing the release of IL-1β and -18, and triggering pyroptosis, a lytic form of cell death that amplifies the inflammatory response [12]. NLRP1 is activated by viral 3 C proteases [13], double stranded RNA generated upon viral infection [14], and by inhibition of binding to dipeptidyl peptidase 8/9 [15] or to oxidized thioredoxin [16], which restrain NLRP1 activation. Moreover, it is has long been known that UVB activates NLRP1 in human keratinocytes [17], which is believed to underlie the induction of sunburn [18]. However, only recently, it was demonstrated that this is regulated by phosphorylation of NLRP1 induced by activation of the ribotoxic stress response downstream of UVB radiation [4, 19].

Although NLRP1 activation in keratinocytes promotes the development of cSCCs, expression of NLRP1 and other inflammasome proteins including IL-1β is strongly suppressed in cSCCs and SCC cell lines [20]. In SCC cell lines, this is partially regulated by promoter methylation [20]. Furthermore, expression of p62 (*SQSTM1*) is strongly induced in cSCCs and suppression of p62 expression rescues UVB-induced NLRP1 activation in SCC cell lines by increasing NLRP1 and IL-1β expression [21]. p62 is a multifunctional protein and a cargo receptor in autophagy [22]. Expression of p62 is induced in several types of cancer playing key roles in cancer development. For example, in hepatocellular carcinoma (HCC), p62 supports carcinogenesis through activation of Nrf2 [23], in papillary thyroid cancer through mTOR activation [24] or in lung adenocarcinomas through NF-κB activation [25]. By its role as cargo receptor, p62 antagonizes the inflammasome pathway through autophagy-dependent degradation of ubiquitinated inflammasome proteins [26, 27] and through targeting of damaged mitochondria, which induce NLRP3 inflammasome activation, for mitophagy [28]. However, in SCC cells, NLRP1 and IL-1β expression are not only suppressed at the protein but also at the mRNA level, which makes a regulation only by autophagy unlikely [21].

MicroRNAs (miRNAs, miRs) are small, non-coding RNAs that regulate gene expression at the mRNA level [29, 30]. They primarily bind to the 3’ untranslated region (3’UTR) of mRNAs via a short seed sequence leading to mRNA degradation, translational inhibition, or, in some cases, increased expression. Additionally, they can be sequestered through a complex physical interactions, making them unavailable for binding to other RNAs (sponging) [31]. miRNAs bind many target RNAs and can fine-tune complex pathways, particularly in cancer development, by a crosstalk between coding and non-coding RNAs, including long non-coding RNAs (lncRNAs) and circular RNAs (circRNAs) [32].

miR-34a is a tumor suppressor in different types of cancer and has been implicated in the regulation of apoptosis, proliferation and metastasis [33, 34]. This miR has over 700 predicted or validated targets, which are involved in critical signaling pathways required for cancer development [33, 34]. Most importantly, expression of miR-34a is regulated by TP53 as well as vice versa and this positive feedback loop suppresses tumor formation by regulating cell cycle arrest, DNA repair, and apoptosis. Particularly, by targeting the negative TP53 regulators SIRT1 and MDM4, miR-34a-5p stabilizes TP53 and enhances the TP53 signaling pathways [35, 36]. Expression of miR-34a-5p is suppressed in cSCCs and SCC cell lines compared to healthy skin or HaCaT keratinocytes, respectively, suggesting that it plays also a role in the development of keratinocyte-derived skin cancer [37].

Here, we identified an inverse regulation of NLRP1 expression by the mRNA of p62 in SCC cells. miR-34a-5p is a mediator of this crosstalk and a novel positive regulator of NLRP1 expression further supporting their importance in the development of cSCCs.

## Results

### The mRNA of p62 regulates NLRP1 activation and expression

Expression of NLRP1 inflammasome proteins is strongly reduced in SCC cell lines compared to human primary keratinocytes (HPKs), as well as in cSCCs compared to healthy skin [20, 21]. This suggests that development of cSCCs is supported by NLRP1 activation at early stages of tumor development [8], but has negative consequences for established cSCCs. Recently, we identified an NF-κB-mediated increase of p62 expression in cSCC and SCC cell lines, which antagonizes the NLRP1 pathway [21]. A knockdown of p62 expression in SCC12 cells or human primary keratinocytes (HPKs) rescued or increased UVB-induced NLRP1 activation, respectively, which is most likely mediated by an increase in NLRP1 and IL-1β expression [21]. The molecular mechanisms underlying suppression of expression of NLRP1 by p62 have not been characterized yet. However, pathways downstream of p62, such as autophagy, mTORC1 or Nrf2 activation, are not involved [21].

**Fig. 1:**
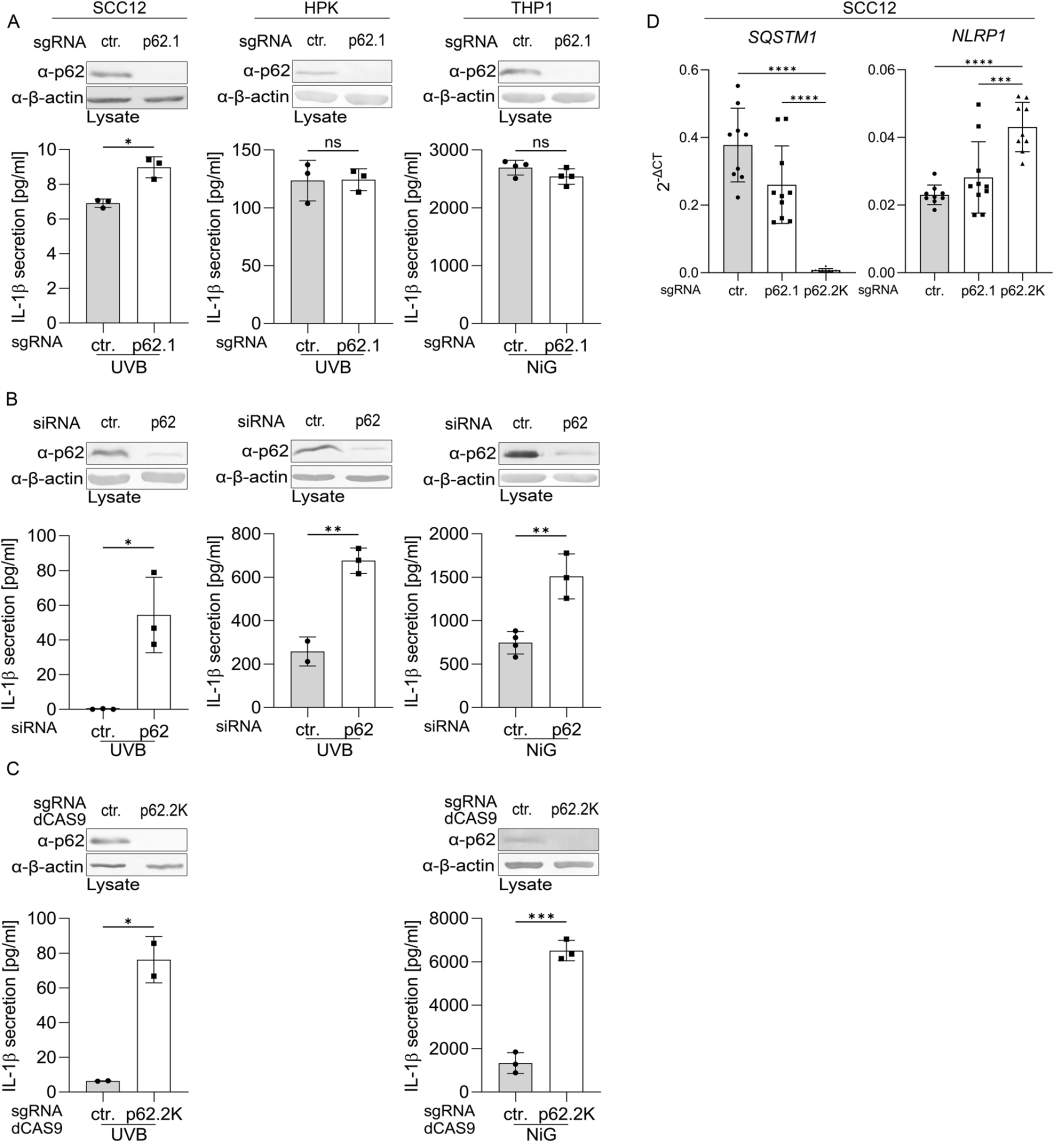
p62 mRNA, rather than protein, regulates inflammasome activation and expression of NLRP1. **A**–**D** Expression of p62 was suppressed by different techniques to assess its impact on NLRP1 inflammasome activation. p62 protein expression was determined by western blot and IL-1β secretion, reflecting inflammasome activation, by ELISA 6 h after UVB irradiation (HPKs and SCCs) or nigericin treatment (THP-1 cells). **A** CRISPR/Cas9 knockout (p62.1 - protein only) of p62, (**B**) siRNA-mediated knockdown (mRNA and protein), or (**C**) CRISPR/dCas9-KRAB knockout (p62.2 K - mRNA and protein) in SCC12 cells, HPKs and THP-1 cells. **D** mRNA levels of NLRP1 and SQSTM1 (p62) upon protein (p62.1) or mRNA/protein (p62.2 K) knockout in SCC12 cells determined by qPCR in mock-treated cells. Data are represented as mean ± SD of three experiments using two-tailed unpaired t-test (*N* = 3) (**A**–**C**) or one-way analysis of variance with Dunnett’s multiple comparison test (*N* = 10) (**D**). (****P < 0.001, ***P ≤ 0.001, **P ≤ 0.01, and *P ≤ 0.05, ns = not significant). SCC squamous cell carcinoma, HPK human primary keratinocyte, THP-1 monocyte cell line.

To address the role of p62 in the regulation of NLRP1 expression, we suppressed p62 expression in SCC12 cells by three different experimental approaches, namely classical CRISPR/Cas9, siRNA, and CRISPR/dCas9. Then, the cells were irradiated with a physiological dose of UVB and analyzed for NLRP1 inflammasome activation through quantification of mature IL-1β in the supernatant (Fig. 1A–C). Whereas a p62 knockdown and dCas9 knockout rescued IL-1β secretion and, therefore, NLRP1 activation, this was not the case when p62 expression was suppressed by a classical Cas9 knockout. We obtained similar results with UVB-irradiated HPKs or NLRP3 activated THP-1 cells. Again, only a knockdown or dCas9 knockout, but not a Cas9 knockout, significantly increased IL-1β secretion, although p62 protein expression was efficiently suppressed by all approaches (Fig. 1A–C). While siRNA induces degradation of its target mRNA and dCas9 blocks mRNA transcription by binding to the promoter, CRISPR/Cas9 typically disrupts protein expression by altering the reading frame and introducing a premature stop codon, often leaving the level of the altered mRNA unaffected. Indeed, a classical CRISPR/Cas9 knockout in SCC12 cells reduced p62 mRNA only slightly, whereas dCas9 blocked it completely (Fig. 1D). Most importantly, p62 and NLRP1 mRNA expression were correlated in an inverse manner (Fig. 1D), suggesting that p62 mRNA regulates NLRP1 mRNA in a negative manner.

### p62 mRNA regulates expression of mRNAs and miRs

**Fig. 2:**
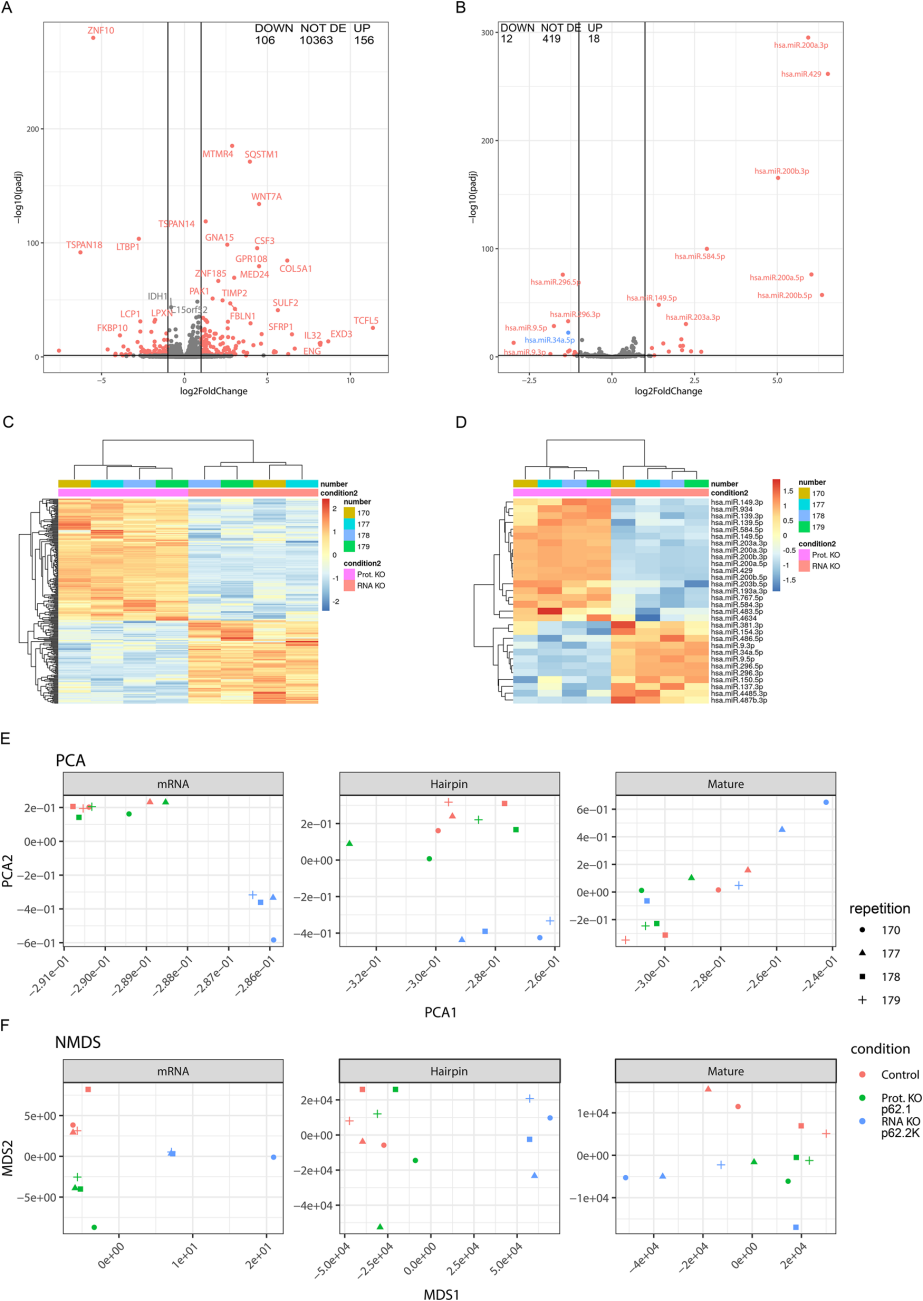
p62 mRNA, but not protein, regulates expression of other mRNAs and miRNAs. **A–D** RNA sequencing data showing influence of p62 mRNA knockout on gene (**A, C**) and miRNA (**B, D**) expression. Differential expression analysis of RNA-seq data from SCC12 cells with a protein (p62.1) or mRNA (p62.2 K) knockout represented by Volcano plot (each dot represents one gene) and Heatmap showing differentially expressed genes or mature miRNAs. **E** Principal component analysis (PCA) and (**F**) non-metric multidimensional scaling (NMDS) (blue for RNA knockout, red and green for control and protein only knockout, respectively), highlighting distinct gene expression patterns. DOWN (downregulated genes/ miRNAs), NOT DE (mRNA/miRNA that were not significantly regulated in the screen), UP (upregulated genes/ miRNAs); *N* = 4.

For the elucidation of the molecular mechanisms underlying the regulation of NLRP1 by p62 mRNA, we performed an RNA sequencing experiment in SCC12 cells. Upon lentiviral transduction and selection with antibiotics, we generated stable polyclonal dCas9 control, p62 protein (by Cas9) and p62 mRNA/protein (by dCas9) knockout SCC12 cells. Total RNA was isolated (quadruplet) and analyzed by RNA sequencing (Fig. 2). We identified 156 or 106 genes (mRNAs), which were up- or down-regulated by p62 mRNA, respectively (Fig. 2A, Supplementary CSV File). Furthermore, 30 miRs were regulated by p62 mRNA, from which 18 were up- and 12 downregulated (Fig. 2B and Supplementary Fig. 1). Interestingly, several miRs were suppressed by a p62 mRNA knockout and, therefore, their expression is positively correlated with NLRP1 expression. This experiment indeed demonstrates that p62 mRNA is able to regulate mRNA and miR expression in a positive as well as in a negative manner.

### Validation of p62 mRNA-dependent gene expression

**Fig. 3:**
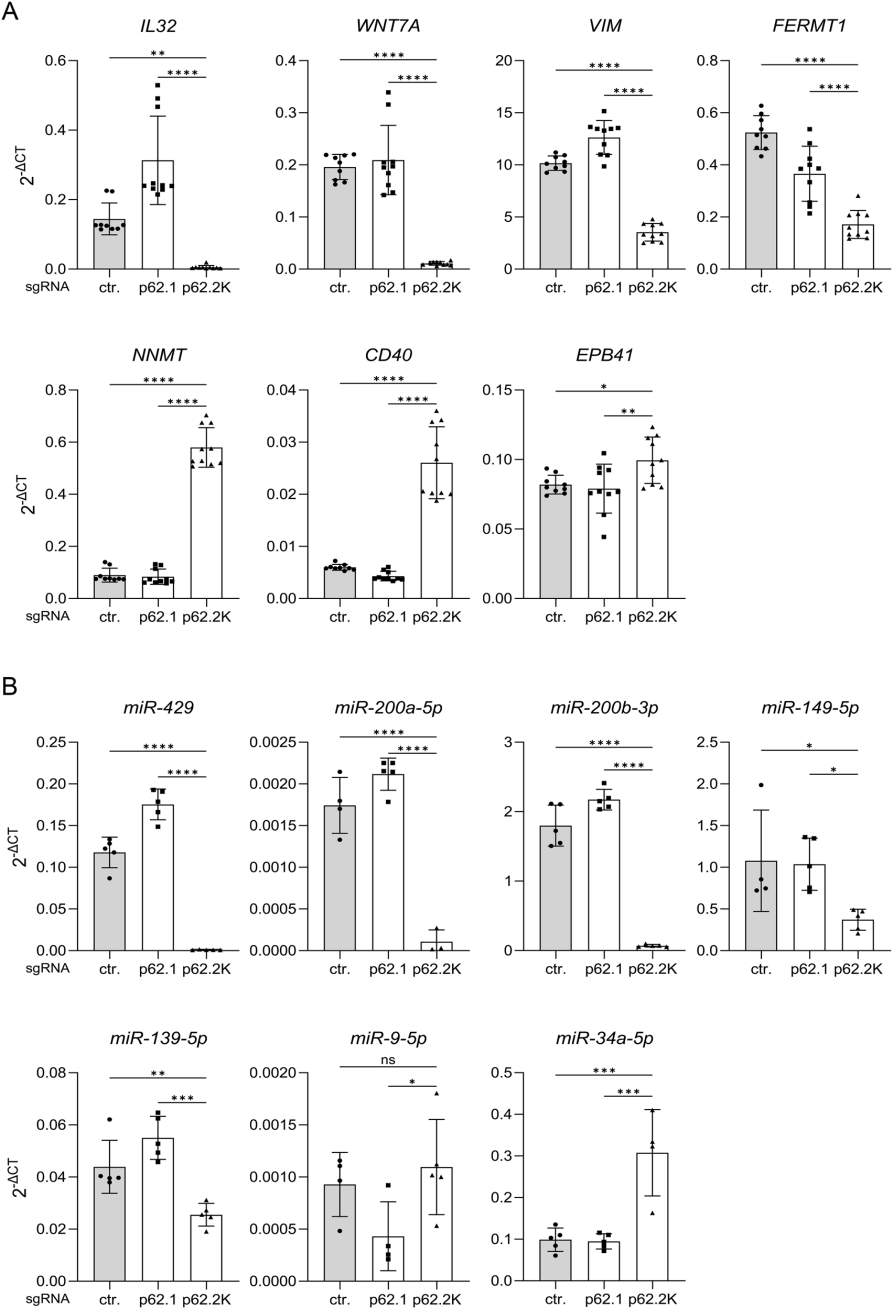
Validation of p62 mRNA-regulated genes (A) and miRNAs (B) using qPCR. **A, B** SCC12 cells were transduced with a control (ctr.) gRNA or two different sgRNAs targeting p62 protein (p62.1), or mRNA (p62.2 K). Levels of mRNA (**A**) of the indicated genes and miRNAs (**B**) in mock-treated cells were determined by qPCR. Data are represented as mean ± SD of *N* = 10 (**A**) and *N* = 5 (**B**), p values are visualized using one-way analysis of variance with Dunnett’s multiple comparison test. (****P < 0.001, ***P ≤ 0.001, **P ≤ 0.01, and *P ≤ 0.05, ns = not significant).

For the validation of the RNA sequencing experiment, we chose genes and miRs, whose expression was strongly regulated by the p62 mRNA and only to a minimal extent by the p62 protein (not shown, Supplementary Fig. 1, Supplementary CSV File). Several identified genes are involved in tumorigenesis, including in the development of cSCCs, and particularly in epithelial-mesenchymal transition (EMT), such as WNT7A, VIM, or FERMT1 [38–40] (Fig. 3A). Interestingly, we identified a suppression of miR-149-5p expression by a p62 mRNA knockout (Fig. 3B). In nucleus pulposus cells, this miR is a confirmed negative regulator of NLRP1 expression through binding to its mRNA by the seed sequence [41]. However, in SCC12 cells we could not confirm a regulation of NLRP1 expression by miR-149 (not shown). In contrast, a p62 mRNA knockout induced expression of miR-34a-5p (Fig. 3B), suggesting that miR-34a-5p expression is positively correlated with NLRP1 expression. miR-34a-5p is directly regulated by TP53, an established tumor suppressor in different types of cancer, which expression is also suppressed in cSCCs [34, 37, 42]. In summary, we could confirm by independent experiments, the regulations of mRNAs and miRs through p62 mRNA, as suggested by the RNA sequencing screen.

### miR-34a-5p is a novel positive regulator of NLRP1 expression

**Fig. 4:**
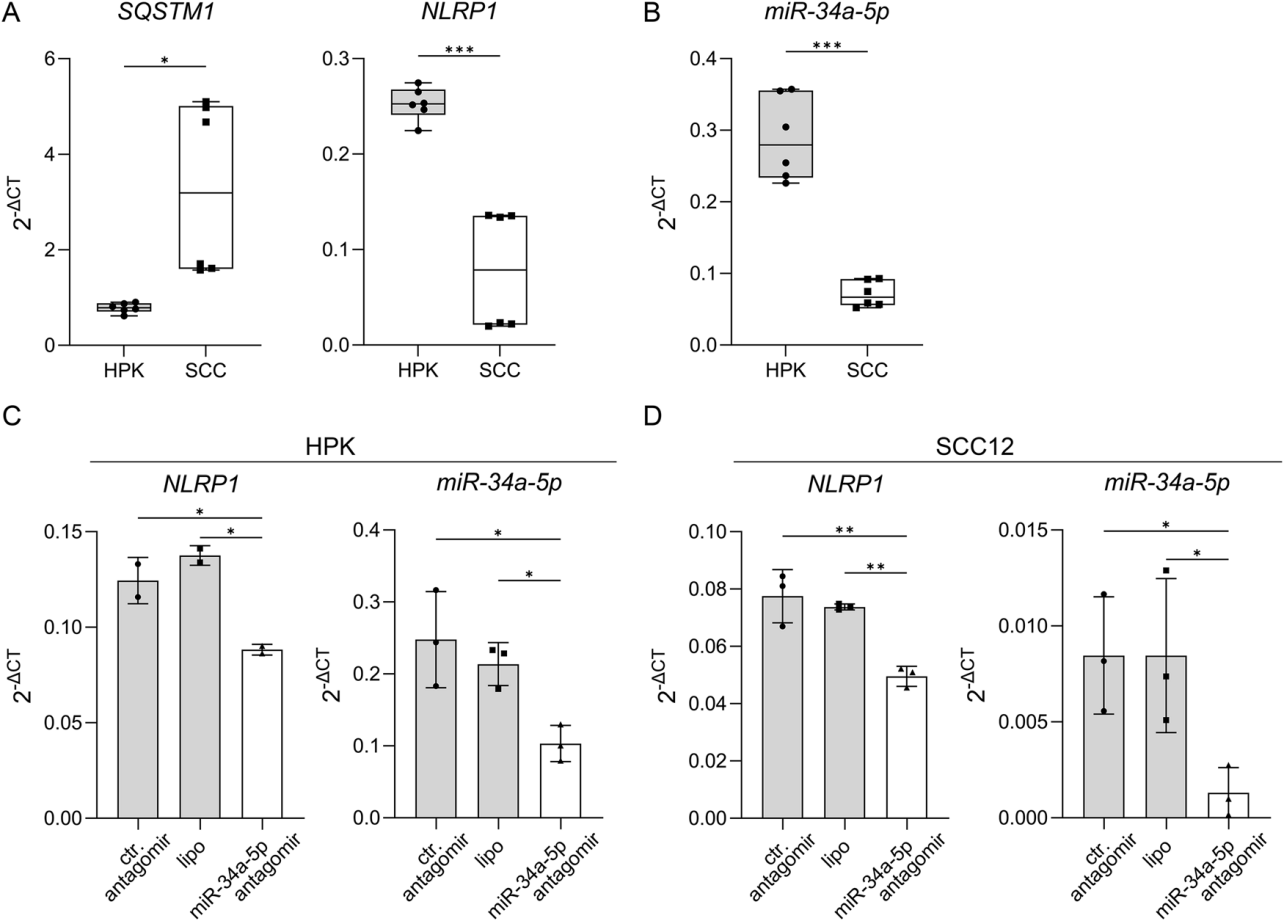
miR-34a-5p regulates NLRP1 expression. **A**, **B** HPKs from two different donors were compared with SCC12 and SCC13 cells. **A** mRNA levels of SQSTM1 (p62), NLRP1, or (**B**) miRNA34a-5p were determined via qPCR. (**C, D**) HPKs (*N* = 2) and SCC12 cells (*N* = 3) were transfected with control (ctr.) antagomir or miR-34a-5p antagomir or treated with lipofectamine only, and analyzed using qPCR. **A–D** Data are represented as mean ± SD of three experiments (**A**, **B**) where two donors in the group of HPKs (*N* = 6) and two SCC cell lines (SCC12, SCC13) in the group of SCCs (*N* = 6) were subjected to two-tailed unpaired t-test with Welch’s correction or (**C, D**) one-way analysis of variance with Dunnett’s multiple comparison test. (****P < 0.001, ***P ≤ 0.001, **P ≤ 0.01, and *P ≤ 0.05, ns = not significant). SCC squamous cell carcinoma, HPK human primary keratinocyte, lipo lipofectamine.

Then, we characterized expression of mRNA of p62 and NLRP1 and of miR-34a-5p in HPKs from different donors and in SCC cell lines (Fig. 4A, B). As expected [37], expression of miR-34a-5p is strongly reduced in SCC cells lines demonstrating a positive correlation with NLRP1. To test, whether this correlation is functionally relevant, we transfected miR-34a-5p antagomir into HPKs and SCC12 cells, control antagomir and lipofectamine only served as controls (Fig. 4C, D). Surprisingly, a suppression of miR-34a-5p reduced NLRP1 expression demonstrating that miR-34a-5p indeed regulates expression of the inflammasome sensor in a positive manner. Most likely, this occurs through an indirect mechanism, because miR-34a-5p, although it has about 700 potential target mRNAs, cannot target NLRP1 mRNA directly [34, 41] (not shown).

## Discussion

**Fig. 5:**
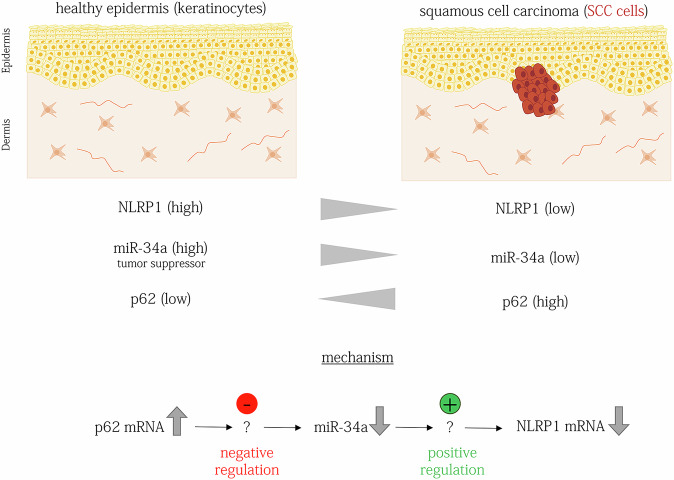
Graphical Summary. In healthy skin, keratinocytes express high levels of NLRP1 and of the tumor suppressor miR-34a, while p62 expression remains relatively low. In cutaneous squamous cell carcinoma (cSCC) tumor cells, the expression of NLRP1 and miR-34a is decreased, while p62 expression is elevated. p62 mRNA negatively regulates miR-34a, while miR-34a positively regulates NLRP1 mRNA expression. Consequently, the increased expression of p62 in SCC cells leads to the suppression of NLRP1 expression, mediated by a decrease in miR-34a. Most likely, miR-34a does directly interact neither with p62 mRNA nor with NLRP1 mRNA.

In this study, we analyzed the molecular mechanisms underlying suppression of NLRP1 expression in cSCC and SCC cell lines by p62/*SQSTM1*. We identified a regulation at the RNA level and identified miR-34a-5p as a novel positive regulator of NLRP1 mRNA expression in human keratinocytes and SCC cells downstream of p62 mRNA (Fig. 5 Graphical Summary). The p62 mRNA is interconnected with RNA expression of several genes and miRs involved in cancer development. In addition to the known mechanisms, how the p62 protein supports cancer development in different tissues, the negative regulation of NLRP1 expression by p62 mRNA mediated by miR-34a-5p represents a novel pathway in the development of cSCCs. Furthermore, the p62 regulation of miR-34a-5p, which is an established tumor suppressor in different types of cancer, might be also relevant for carcinogenic conversion in other tissues beyond the skin.

Aberrant and chronic activation of NLRP1 predisposes patients for the development of cSCCs demonstrating that NLRP1 is a tumor promoter [8]. However, expression of the NLRP1 inflammasome is suppressed in cSCCs and cell lines, suggesting that the NLRP1 pathway has negative effects for SCC cells [20]. Indeed, NLRP1 ablation in SCC cells increases their stress resistance [21]. It is also possible that suppression of NLRP1 expression in cSCCs protects the cancer cells from anti-tumor immunity induced by NLRP1 activation. However, this is difficult to address experimentally, as the NLRP1 pathway in human skin is only poorly conserved in mice [43]. Aldara, a cream for the treatment of BCCs, atopic keratosis, and SCC in situ, induces NLRP1 inflammasome activation and pyroptosis in HPKs [44]. However, Aldara is not efficient for the treatment of cSCCs, where NLRP1 expression is suppressed. Therefore, it is tempting to speculate that NLRP1 activation contributes to the pharmacological activity of Aldara.

Interestingly, we demonstrated recently an upregulation of p62 expression in cSCCs, but not in BCCs, which is responsible for NLRP1 suppression, further suggesting a role of NLRP1 activation in the pharmacological efficacy of Aldara [21]. p62 expression is upregulated in several types of cancer with in part key roles in carcinogenic conversion [22]. It acts not only as a cargo receptor in autophagy, but is a multifunctional protein affecting several cellular key regulators in cancer development, including Nrf2, NF-κB, or mTOR. However, inhibition of NLRP1 expression by p62 in SCC cell lines is not mediated by the known pathways regulated by p62 [21].

In this study, we identified a regulation of expression of the mRNAs of NLRP1 and several other genes and of miRs through the p62 mRNA (Figs. 1, 2). Usually, genes exert their function at the level of proteins. However, miRs, lncRNAs, pseudo genes, and circRNAs are not translated into protein, but are regulators at the RNA level. Together with mRNAs, they are linked through a partial sequence complementarity between microRNA response elements (MREs) and the seed sequence of miRs, forming a complex and dynamic network. lncRNAs and circRNAs can bind miRs, and thereby inactivate and “sponge” them, which is particularly significant in cancer development [45, 46]. This regulation can be extremely complex, because miRs usually interact with many transcripts, whereas mRNAs can harbor several MREs [32]. It is also discussed that the interactions of miRs with other RNAs including mRNAs are bidirectional meaning that the single RNAs of a pool competing for a given miR are linked and thereby regulate their activity, termed competing endogenous RNAs (ceRNAs) [32]. This might explain how p62 mRNA regulates the levels of several other mRNAs and miRs (Fig. 2).

NLRP1 expression is regulated by several miRs [47–52], which are partially known to interact with lncRNAs, such as miR-637 or miR-149-5p [41, 53]. Although we identified a regulation of miR-149-5p by p62 mRNA (Fig. 3), we could not confirm a regulation of NLRP1 by miR-149-5p in SCC12 cells. In contrast to miR-149-5p, which acts as a tumor suppressor or oncogene [54], miR-34a-5p acts always as tumor suppressor in different types of cancer, including cSCC [34, 37]. Although neither NLRP1 nor p62 are direct targets of miR-34a-5p [33], a knockout of p62 mRNA causes miR-34a-5p upregulation (Fig. 3 and Supplementary Fig. S1) and antagomir-induced suppression of its expression downregulates NLRP1 mRNA (Fig. 4). Therefore, miR-34a-5p is an indirect positive regulator of NLRP1 mRNA expression suggesting that, in the simplest case, it negatively regulates an unknown miR that suppresses NLRP1 mRNA expression. In SCC cells, miR-34a-5p inhibits proliferation and migration, and increases apoptosis [37]. In other cancers, miR-34a-5p is discussed as a prognostic marker as well as a potential pharmacological target, which low expression is associated with a poor prognosis of cancer patients. Supplementation with chemically synthesized, particularly stable miR-34a-5p mimics is a promising novel approach for the treatment of cancers characterized by suppression of miR-34a-5p expression [33, 34]. This is particularly true for cSCCs, which can be treated by topical approaches. Our finding of a positive regulation of NLRP1 expression by miR-34a raises the possibility that pharmacological treatment of cSCC patients with mimics of the miR might increase anti-tumor immunity and other tumor suppressive effects downstream of NLRP1 [21]. Furthermore, miR-34a-5p silences several proto-oncogenes, inhibits EMT and targets genes regulating the cell cycle [33, 34]. A complex regulation of miR-34a-5p expression through sponging by different lncRNAs has been described [33]. TP53, which is frequently inactivated in cancer, including in cSCCs, directly regulates expression of miR-34a-5p [33]. Our finding of a regulation of miR-34a-5p expression by p62 mRNA in HPKs and SCC cells suggests that p62 might also contribute to cancer development by suppression of miR-34a-5p in other tissues, as p62 upregulation is a common event in carcinogenesis [22].

In summary, we identified a novel pathway at the RNA level with potential relevance for the development and treatment of cSCCs, but also of other types of tumors, namely the regulation of NLRP1 mRNA expression through p62 mRNA, which is mediated by miR-34a-5p, an established tumor suppressor in different tissues. As miR-34a-5p cannot directly interact with the mRNAs of p62 or NLRP1, more research is needed to fully elucidate the molecular mechanisms underlying this pathway.

## Materials and Methods

### Culture and treatment of primary cells and SCC cell lines

Isolation and culture of human primary keratinocytes (HPKs) was performed as described previously [55]. HPKs and SCC12 were grown in serum-free keratinocyte medium (KSFM, Thermo Fisher Scientific, USA) supplemented with epidermal growth factor (EGF) and bovine pituitary extract (BPE). SCC13 were cultured in Dulbecco’s Modified Eagle’s Medium (DMEM, Gibco, USA) supplemented with 10% fetal bovine serum FBS (PAN-Biotech, Germany). THP-1 cells (ATCC TIB-202) were cultivated in RPMI 1640 (DMEM, Gibco, USA), supplemented with 10% FBS, 1% A/A, sodium pyruvate (1 mM) (Gibco, USA), 1% GlutaMax (Gibco, USA).

Cells were harvested in trypsin/EDTA solution (0.05%/0.02% w/v) (Thermo Fisher Scientific) and cultured for at least 48 h before experiments. All cells were incubated at 37 °C in 5% CO_2_ and 95% humidity. HPKs and SCCs were stimulated with 0.0875 J/cm^2^ of UVB (UV802L; Waldmann, Villingen-Schwenningen, Germany). THP-1 cells were differentiated with PMA (27 nM) for 3 days, primed with ultra-pure LPS (100 ng/ml; InvivoGen) overnight, and stimulated with 5 µM Nigericin (Selleckchem, Houston, TX). For siRNA-mediated knockdown, cells were transfected with 10 nM siRNAs (Sigma-Aldrich, USA, Supplementary Table 1) using Interferin (Polyplus, France) as a transfection reagent.

All cell lines were authenticated (Microsynth, Switzerland) and were verified to be mycoplasma negative.

### Human biopsies

Isolation of HPKs from skin biopsy samples was performed as described previously [55, 56].

The skin biopsies were collected with informed written consent upon approval from local ethical committees (KEK-ZH-Nr. 2015-0198 and BASEC-Nr. 2024-01030) and were conducted according to the Declaration of Helsinki Principles.

### Generation of CRISPR/Cas9- and CRISPR/dCas9-KRAB-targeted SCCs, THP-1, HPKs

Single-stranded DNA oligonucleotides were designed on the Benchling platform (https://benchling.com) and purchased from Microsynth (Balgach, Switzerland). sgRNAs (Supplementary Table 2, Microsynth, Switzerland) were cloned into the lentiCRISPRv2 plasmid (#52961, Addgene) [57] to generate CRISPR/Cas9-targeted cells. sgRNAs targeting the promoter region were cloned into the pLV hU6-sgRNA hUbC-dCas9-KRAB-T2a-Puro (#71236, Addgene) [58] to generate CRISPR/dCas9-KRAB-targeted cells. Specific plasmids were co-transfected into HEK 293 T cells with the envelope and packaging plasmids psPAX2 (#12260, Addgene) and pMD2.G (#12259, Addgene). After 48 h, lentiviruses were harvested and concentrated by centrifugation (16,000 g, 4 h, 4 °C). The SCC12 cell line and THP-1 cells were transduced with virus resuspended in KSFM or RPMI, respectively, containing 2.5 μg/ml polybrene (hexadimethrine bromide) (Sigma-Aldrich, USA). Transduction with non-targeting empty vector served as a control. Medium was changed 24 h after transduction to KSFM medium containing supplements or RPMI. After transduction, cells were selected with 5 µg/ml puromycin (Sigma-Aldrich, USA) and expanded. Transduction and selection of HPKs was performed in co-culture with antibiotic-resistant 3T3-J2 feeder cells as already described [55, 56]. Efficiency was assessed at the protein level by western blot and on RNA level by qPCR.

### Real-time qPCR

Levels of mRNA were determined by quantitative real-time PCR using the LightCycler 480 instrument and FastStart Essential DNA Green Master (Roche, Switzerland) and specific primers (Microsynth, Switzerland, Supplementary Table 3). For preparation of cDNA from total RNA, 1.5-2 μg of RNA were subjected to the RevertAid First Strand cDNA synthesis kit (Thermo Fisher Scientific). mRNA levels were normalized to HPRT and visualized using 2^−ΔCT^ method.

To determine the miRNA levels total RNA was isolated using the miRNeasy Kit (217004, Qiagen) according to the manufacturer’s instruction. Total RNA (250 ng) was used to generate cDNA using the TaqMan™ Advanced miRNA cDNA Synthesis Kit (A28007, Thermo Fisher Scientific) according to the manufacturer’s instructions. Real-time polymerase chain reaction (qPCR) was performed using the TaqMan™ Advanced miRNA Assay (A25576, Thermo Fisher Scientific, Supplementary Table 3) in the reaction volume of 10 µl on the LightCycler 480 instrument using parameters from table 8 of manufacturer’s protocol. miRNA levels were normalized to endogenous control has-miR-26a-5p and visualized using 2^−ΔCT^ method.

### RNA sequencing

Total RNA was isolated using the miRNeasy Mini Kit (217004, Qiagen). For mRNA sequencing, libraries were prepared with TruSeq RNA (Illumina), while miRNA libraries utilized RealSeq®-AC (somagenics), following standard protocols at the Functional Genomics Centre Zurich (FGCZ). High Throughput Sequencing (NGS) was performed on Illumina Novaseq 6000. Raw reads were quality-checked using FastQC.

mRNA reads were mapped to the human transcriptome using salmon version 1.9.0 [59]. The quantified transcripts were imported into R and collapsed to the gene level using the R package “tximport” [60]. Mature and hairpin microRNA quantification was performed using the nf-core/smrnaseq pipeline v2.3.0 (10.5281/zenodo.3456879 [61]). Differential expression analysis of mRNA and miRNA was performed using the R package DESeq2 [62]. The data discussed in this publication have been deposited in NCBI’s Gene Expression Omnibus [63] and are accessible through GEO Series accession number GSE294764 (https://www.ncbi.nlm.nih.gov/geo/query/acc.cgi?acc=GSE294764).

### miR-34a-5p antagomiR transfection

Cells were seeded two days before to be confluent of ca. 80% on the day of transfection with Lipofectamine 2000 (Invitrogen) (2.5 ul per well of 12 well plate) and 75 nM of antagomir (HY-RI00714A hsa-miR-34a-5p antagomir, MedChemExpress)/ control (HY-RI04602A MicroRNA Antagomir Negative Control MedChemExpress). Only Lipofectamine transfection was also used as an additional negative control. Cells were harvested after 48 h for RNA.

### Immunoblotting

Cell culture lysates were harvested with SDS loading buffer and subjected to sonification. Proteins were separated by SDS-PAGE and analyzed by immunoblotting as previously described. The primary and secondary antibodies used are specified in Supplementary Table 4.

Full and uncropped western blots are included in the supplementary part.

### ELISA

Release of human IL-1β to the supernatant was measured by ELISA (R&D Systems, USA) according to manufacturer’s instruction, and normalized to lactate dehydrogenase (LDH) from corresponding cell lysates obtained from the CytoTox 96 nonradioactive cytotoxicity assay (Promega) according to manufacturer’s instruction.

### Statistical analysis

Statistical analysis of not high throughput data was performed using the GraphPad Prism 9 (GraphPad Software, La Jolla, US-CA). For comparisons of two groups, two-tailed unpaired/ paired t-test. Comparisons of three or more groups were performed using one-way analysis of variance with Dunnett’s post-hoc test. Data are presented as means ± SD. Differences were considered significant when P values were below 0.05 (∗∗∗∗*P* < 0.001, ∗∗∗*P* ≤ 0.001, ∗∗*P* ≤ 0.01, and ∗*P* ≤ 0.05, ns = not significant).

## Supplementary information

Supplementary tables

Figure S1

Legend for Supplementary Figure Fig. S1

Supplementary CSV File

## Data Availability

All datasets generated and analyzed during this study are included in this published article and its supplementary information files. The RNA sequencing data discussed in this publication have been deposited in NCBI’s Gene Expression Omnibus and are accessible through GEO Series accession number GSE294764. Additional data are available from the corresponding author on reasonable request.

## References

[1] Nestle FO, Di Meglio P, Qin JZ, Nickoloff BJ. Skin immune sentinels in health and disease. Nat Rev Immunol. 2009;9:679–91. 19763149 10.1038/nri2622 PMC2947825

[2] Ratushny V, Gober MD, Hick R, Ridky TW, Seykora JT. From keratinocyte to cancer: the pathogenesis and modeling of cutaneous squamous cell carcinoma. J Clin Invest. 2012;122:464–72. 22293185 10.1172/JCI57415 PMC3266779

[3] Molho-Pessach V, Lotem M. Ultraviolet radiation and cutaneous carcinogenesis. Curr Probl Dermatol. 2007;35:14–27. 17641487 10.1159/000106407

[4] Robinson KS, Toh GA, Rozario P, Chua R, Bauernfried S, Sun Z, et al. ZAKalpha-driven ribotoxic stress response activates the human NLRP1 inflammasome. Science. 2022;377:328–35. 35857590 10.1126/science.abl6324 PMC7614315

[5] Bernard JJ, Gallo RL, Krutmann J. Photoimmunology: how ultraviolet radiation affects the immune system. Nat Rev Immunol. 2019;19:688–701. 31213673 10.1038/s41577-019-0185-9

[6] Thompson AK, Kelley BF, Prokop LJ, Murad MH, Baum CL. Risk factors for cutaneous squamous cell carcinoma recurrence, metastasis, and disease-specific death: a systematic review and meta-analysis. JAMA Dermatol. 2016;152:419–28. 26762219 10.1001/jamadermatol.2015.4994 PMC4833641

[7] Fenini G, Karakaya T, Hennig P, Di Filippo M, Slaufova M, Beer HD. NLRP1 inflammasome activation in keratinocytes: increasing evidence of important roles in inflammatory skin diseases and immunity. J Invest Dermatol. 2022;142:2313–22. 35550825 10.1016/j.jid.2022.04.004

[8] Zhong FL, Mamai O, Sborgi L, Boussofara L, Hopkins R, Robinson K, et al. Germline NLRP1 mutations cause skin inflammatory and cancer susceptibility syndromes via inflammasome activation. Cell. 2016;167:187–202 e117. 27662089 10.1016/j.cell.2016.09.001

[9] Broz P, Dixit VM. Inflammasomes: mechanism of assembly, regulation and signalling. Nat Rev Immunol. 2016;16:407–20. 27291964 10.1038/nri.2016.58

[10] Barnett KC, Li S, Liang K, Ting JPA. 360 degrees view of the inflammasome: mechanisms of activation, cell death, and diseases. Cell. 2023;186:2288–312. 37236155 10.1016/j.cell.2023.04.025 PMC10228754

[11] Lillo S, Saleh M. Inflammasomes in cancer progression and anti-tumor immunity. Front Cell Dev Biol. 2022;10:839041. 35517498 10.3389/fcell.2022.839041 PMC9065266

[12] Broz P, Pelegrin P, Shao F. The gasdermins, a protein family executing cell death and inflammation. Nat Rev Immunol. 2020;20:143–57. 31690840 10.1038/s41577-019-0228-2

[13] Robinson KS, Teo DET, Tan KS, Toh GA, Ong HH, Lim CK, et al. Enteroviral 3C protease activates the human NLRP1 inflammasome in airway epithelia. *Science* 2020;370. 10.1126/science.aay2002 33093214

[14] Bauernfried S, Scherr MJ, Pichlmair A, Duderstadt KE, Hornung V. Human NLRP1 is a sensor for double-stranded RNA. Science 2021;371. 10.1126/science.abd0811 33243852

[15] Zhong FL, Robinson K, Teo DET, Tan KY, Lim C, Harapas CR, et al. Human DPP9 represses NLRP1 inflammasome and protects against autoinflammatory diseases via both peptidase activity and FIIND domain binding. J Biol Chem. 2018;293:18864–78. 30291141 10.1074/jbc.RA118.004350 PMC6295727

[16] Ball DP, Tsamouri LP, Wang AE, Huang HC, Warren CD, Wang Q, et al. Oxidized thioredoxin-1 restrains the NLRP1 inflammasome. Sci Immunol. 2022;7:eabm7200. 36332009 10.1126/sciimmunol.abm7200 PMC9850498

[17] Feldmeyer L, Keller M, Niklaus G, Hohl D, Werner S, Beer HD. The inflammasome mediates UVB-induced activation and secretion of interleukin-1beta by keratinocytes. Curr Biol. 2007;17:1140–5. 17600714 10.1016/j.cub.2007.05.074

[18] Faustin B, Reed JC. Sunburned skin activates inflammasomes. Trends Cell Biol. 2008;18:4–8. 18083030 10.1016/j.tcb.2007.10.004

[19] Jenster LM, Lange KE, Normann S, vom Hemdt A, Wuerth JD, Schiffelers LDJ, et al. P38 kinases mediate NLRP1 inflammasome activation after ribotoxic stress response and virus infection. J Exp Med. 2023;220. 10.1084/jem.20220837 PMC9623368 36315050

[20] Sand J, Fenini G, Grossi S, Hennig P, Di Filippo M, Levesque M, et al. The NLRP1 inflammasome pathway is silenced in cutaneous squamous cell carcinoma. J Invest Dermatol. 2019;139:1788–97 e1786. 30738816 10.1016/j.jid.2019.01.025

[21] Hennig P, Di Filippo M, Bilfeld G, Mellett M, Beer HD. High p62 expression suppresses the NLRP1 inflammasome and increases stress resistance in cutaneous SCC cells. Cell Death Dis. 2022;13:1077. 36581625 10.1038/s41419-022-05530-0 PMC9800582

[22] Hennig P, Fenini G, Di Filippo M, Karakaya T, Beer HD. The pathways underlying the multiple roles of p62 in inflammation and cancer. Biomedicines 2021;9. 10.3390/biomedicines9070707 PMC8301319 34206503

[23] Saito T, Ichimura Y, Taguchi K, Suzuki T, Mizushima T, Takagi K, et al. p62/Sqstm1 promotes malignancy of HCV-positive hepatocellular carcinoma through Nrf2-dependent metabolic reprogramming. Nat Commun. 2016;7:12030. 27345495 10.1038/ncomms12030 PMC4931237

[24] Yu F, Ma R, Liu C, Zhang L, Feng K, Wang M, et al. SQSTM1/p62 promotes cell growth and triggers autophagy in papillary thyroid cancer by regulating the AKT/AMPK/mTOR signaling pathway. Front Oncol. 2021;11:638701. 33937040 10.3389/fonc.2021.638701 PMC8082099

[25] Duran A, Linares JF, Galvez AS, Wikenheiser K, Flores JM, Diaz-Meco MT, et al. The signaling adaptor p62 is an important NF-kappaB mediator in tumorigenesis. Cancer Cell. 2008;13:343–54. 18394557 10.1016/j.ccr.2008.02.001

[26] Takahama M, Akira S, Saitoh T. Autophagy limits activation of the inflammasomes. Immunol Rev. 2018;281:62–73. 29248000 10.1111/imr.12613

[27] Shi CS, Shenderov K, Huang NN, Kabat J, Abu-Asab M, Fitzgerald KA, et al. Activation of autophagy by inflammatory signals limits IL-1beta production by targeting ubiquitinated inflammasomes for destruction. Nat Immunol. 2012;13:255–63. 22286270 10.1038/ni.2215 PMC4116819

[28] Zhong Z, Umemura A, Sanchez-Lopez E, Liang S, Shalapour S, Wong J, et al. NF-kappaB restricts inflammasome activation via elimination of damaged mitochondria. Cell. 2016;164:896–910. 26919428 10.1016/j.cell.2015.12.057 PMC4769378

[29] Garzon R, Calin GA, Croce CM. MicroRNAs in Cancer. Annu Rev Med. 2009;60:167–79. 19630570 10.1146/annurev.med.59.053006.104707

[30] Romano G, Veneziano D, Acunzo M, Croce CM. Small non-coding RNA and cancer. Carcinogenesis. 2017;38:485–91. 28449079 10.1093/carcin/bgx026 PMC6248440

[31] Thomson DW, Dinger ME. Endogenous microRNA sponges: evidence and controversy. Nat Rev Genet. 2016;17:272–83. 27040487 10.1038/nrg.2016.20

[32] Ala U. Competing endogenous RNAs, non-coding RNAs and diseases: an intertwined story. Cells 2020;9. 10.3390/cells9071574 PMC7407898 32605220

[33] Li S, Wei X, He J, Cao Q, Du D, Zhan X, et al. The comprehensive landscape of miR-34a in cancer research. Cancer Metastasis Rev. 2021;40:925–48. 33959850 10.1007/s10555-021-09973-3

[34] Fu J, Imani S, Wu MY, Wu RC. MicroRNA-34 family in cancers: role, mechanism, and therapeutic potential. Cancers (Basel) 2023;15. 10.3390/cancers15194723 PMC10571940 37835417

[35] Yamakuchi M, Lowenstein CJ. MiR-34, SIRT1 and p53: the feedback loop. Cell Cycle. 2009;8:712–5. 19221490 10.4161/cc.8.5.7753

[36] Navarro F, Lieberman J. miR-34 and p53: New Insights into a Complex Functional Relationship. PLoS ONE. 2015;10:e0132767. 26177460 10.1371/journal.pone.0132767 PMC4503669

[37] Chen S, Yuan M, Chen H, Wu T, Wu T, Zhang D, et al. MiR-34a-5p suppresses cutaneous squamous cell carcinoma progression by targeting SIRT6. Arch Dermatol Res. 2024;316:299. 38819446 10.1007/s00403-024-03106-w PMC11143063

[38] Kirikoshi H, Katoh M. Expression of WNT7A in human normal tissues and cancer, and regulation of WNT7A and WNT7B in human cancer. Int J Oncol. 2002;21:895–900. 12239632

[39] Dongre A, Weinberg RA. New insights into the mechanisms of epithelial-mesenchymal transition and implications for cancer. Nat Rev Mol Cell Biol. 2019;20:69–84. 30459476 10.1038/s41580-018-0080-4

[40] Scanlon CS, Van Tubergen EA, Inglehart RC, D’Silva NJ. Biomarkers of epithelial-mesenchymal transition in squamous cell carcinoma. J Dent Res. 2013;92:114–21. 23128109 10.1177/0022034512467352 PMC3545688

[41] Li T, Peng Y, Chen Y, Huang X, Li X, Zhang Z, et al. Long intergenic non-coding RNA -00917 regulates the proliferation, inflammation, and pyroptosis of nucleus pulposus cells via targeting miR-149-5p/NOD-like receptor protein 1 axis. Bioengineered. 2022;13:6036–47. 35184666 10.1080/21655979.2022.2043100 PMC8974084

[42] Hermeking H. p53 enters the microRNA world. Cancer Cell. 2007;12:414–8. 17996645 10.1016/j.ccr.2007.10.028

[43] Sand J, Haertel E, Biedermann T, Contassot E, Reichmann E, French LE, et al. Expression of inflammasome proteins and inflammasome activation occurs in human, but not in murine keratinocytes. Cell Death Dis. 2018;9:24. 29348630 10.1038/s41419-017-0009-4 PMC5833864

[44] Walter A, Schafer M, Cecconi V, Matter C, Urosevic-Maiwald M, Belloni B, et al. Aldara activates TLR7-independent immune defence. Nat Commun. 2013;4:1560. 23463003 10.1038/ncomms2566

[45] Zhang W, Xu C, Yang Z, Zhou J, Peng W, Zhang X, et al. Circular RNAs in tumor immunity and immunotherapy. Mol Cancer. 2024;23:171. 39169354 10.1186/s12943-024-02082-z PMC11337656

[46] Bhattacharjee R, Prabhakar N, Kumar L, Bhattacharjee A, Kar S, Malik S, et al. Crosstalk between long noncoding RNA and microRNA in Cancer. Cell Oncol (Dordr). 2023;46:885–908. 37245177 10.1007/s13402-023-00806-9 PMC10356678

[47] Li M, Hu L, Ke Q, Ruan C, Liu X. miR-122-3p alleviates LPS-induced pyroptosis of macrophages via targeting NLRP1. Ann Clin Lab Sci. 2023;53:578–86. 37625833

[48] Wang T, Long Q, Hu Y, Yang Y, Li X, Wei H. miR-181c-5p suppresses neuronal pyroptosis via NLRP1 in Alzheimer’s disease. Behav Brain Res. 2023;447:114387. 37003492 10.1016/j.bbr.2023.114387

[49] Gong H, Wan X, Zhang Y, Liang S. Downregulation of HOTAIR reduces neuronal pyroptosis by targeting miR-455-3p/NLRP1 axis in propofol-treated neurons in vitro. Neurochem Res. 2021;46:1141–50. 33534059 10.1007/s11064-021-03249-6

[50] Chen Z, Dong WH, Chen Q, Li QG, Qiu ZM. Downregulation of miR-199a-3p mediated by the CtBP2-HDAC1-FOXP3 transcriptional complex contributes to acute lung injury by targeting NLRP1. Int J Biol Sci. 2019;15:2627–40. 31754335 10.7150/ijbs.37133 PMC6854378

[51] Cao Y, Zhang H, Lu X, Wang J, Zhang X, Sun S, et al. Overexpression of microRNA-9a-5p ameliorates NLRP1 inflammasome-mediated ischemic injury in rats following ischemic stroke. Neuroscience. 2020;444:106–17. 31954830 10.1016/j.neuroscience.2020.01.008

[52] Gu C, Draga D, Zhou C, Su T, Zou C, Gu Q, et al. miR-590-3p inhibits pyroptosis in diabetic retinopathy by targeting NLRP1 and inactivating the NOX4 signaling pathway. Invest Ophthalmol Vis Sci. 2019;60:4215–23. 31618425 10.1167/iovs.19-27825

[53] Ge JY, Yan XJ, Yang J, Jin H, Sun ZK, Guo JL, et al. LINC00346 regulates NLRP1-mediated pyroptosis and autophagy via binding to microRNA-637 in vascular endothelium injury. Cell Signal. 2023;109:110740. 37268163 10.1016/j.cellsig.2023.110740

[54] Ren FJ, Yao Y, Cai XY, Cai YT, Su Q, Fang GY. MiR-149-5p: an important miRNA regulated by competing endogenous RNAs in diverse human cancers. Front Oncol. 2021;11:743077. 34722295 10.3389/fonc.2021.743077 PMC8554335

[55] Fenini G, Grossi S, Contassot E, Biedermann T, Reichmann E, French LE, et al. Genome editing of human primary keratinocytes by CRISPR/Cas9 reveals an essential role of the NLRP1 inflammasome in UVB sensing. J Invest Dermatol. 2018;138:2644–52. 30096351 10.1016/j.jid.2018.07.016

[56] Grossi S, Fenini G, Hennig P, Di Filippo M, Beer HD. Generation of Knockout Human Primary Keratinocytes by CRISPR/Cas9. Methods Mol Biol. 2019. 10.1007/7651_2019_262 31502220

[57] Sanjana NE, Shalem O, Zhang F. Improved vectors and genome-wide libraries for CRISPR screening. Nat Methods. 2014;11:783–4. 25075903 10.1038/nmeth.3047 PMC4486245

[58] Thakore PI, D’Ippolito AM, Song L, Safi A, Shivakumar NK, Kabadi AM, et al. Highly specific epigenome editing by CRISPR-Cas9 repressors for silencing of distal regulatory elements. Nat Methods. 2015;12:1143–9. 26501517 10.1038/nmeth.3630 PMC4666778

[59] Patro R, Duggal G, Love MI, Irizarry RA, Kingsford C. Salmon provides fast and bias-aware quantification of transcript expression. Nat Methods. 2017;14:417–9. 28263959 10.1038/nmeth.4197 PMC5600148

[60] Soneson C, Love MI, Robinson MD. Differential analyses for RNA-seq: transcript-level estimates improve gene-level inferences. F1000Res. 2015;4:1521. 26925227 10.12688/f1000research.7563.1 PMC4712774

[61] [cited 2024]Available from: https://zenodo.org/records/10696391.

[62] Love MI, Huber W, Anders S. Moderated estimation of fold change and dispersion for RNA-seq data with DESeq2. Genome Biol. 2014;15:550. 25516281 10.1186/s13059-014-0550-8 PMC4302049

[63] Edgar R, Domrachev M, Lash AE. Gene Expression Omnibus: NCBI gene expression and hybridization array data repository. Nucleic Acids Res. 2002;30:207–10. 11752295 10.1093/nar/30.1.207 PMC99122

