# Supplementary material for: p62 mRNA suppresses NLRP1 expression in cutaneous SCC cells through miR-34a-5p
Source: Cell Death Dis. 2025 Jul 1;16(1):465. doi: 10.1038/s41419-025-07785-9 (PMC12218052; doi:10.1038/s41419-025-07785-9)
Supplement: Supplementary file 1 — Supplementary tables [file 41419_2025_7785_MOESM1_ESM.docx]

**Tables Materials and Methods**

**Supplementary table 1 siRNA sequences for siRNA-mediated knockdown**

| **siRNA** | **oligo name** | **sequence 5'-3'** |
| --- | --- | --- |
| Ctr. | Mission siRNA Universal negative control #1 (SIC001) | (Sigma-Aldrich) |
| p62 | SASI_Hs01_00118618 | GGCUGAAGGAAGCUGCCUU [dTdT] |

**Supplementary table 2 Single-stranded DNA sequences for CRISPR/Cas9 and CRISPR/dCas9-KRAB targeting**

| **sgRNA targeting exon** | **sequence 5'-3'** |
| --- | --- |
| control (ctr.) | Forward CACCGGTAGCGAACGTGTCCGGCGT  Reverse AAACACGCCGGACACGTTCGCTACG |
| p62.1 | Forward CACCGGCCTACCTTCTGGGCAAGG  Reverse AAACCCTTGCCCAGAAGGTAGGCC |
| **sgRNA targeting promoter** |  |
| p62.2K | Forward CACCGTGAAGGGGCCTCTGCAGGG  Reverse AAACCCCTGCAGAGGCCCCTTCAC |

**Supplementary table 3 Primers used for Real-time qPCR**

| **Real-time PCR primers** | **sequence 5'-3'** |
| --- | --- |
| *HPRT (housekeeping)* | Forward ATTGTAATGACCAGTCAACAGGG  Reverse GCATTGTTTTGCCAGTGTCAA |
| *NLRP1* | Forward CAGGCAGCACAGATCAACAT  Reverse GTGACCTTGAGGACGGAGAA |
| *SQSTM1/p62* | Forward GACTACGACTTGTGTAGCGTC  Reverse AGTGTCCGTGTTTCACCTTCC |
| *IL32* | Forward TCAAAGAGGGCTACCTGGAGAC  Reverse TCTGTTGCCTCGGCACCGTAAT |
| *VIM* | Forward AGGCAAAGCAGGAGTCCACTGA  Reverse ATCTGGCGTTCCAGGGACTCAT |
| *NNMT* | Forward GTTTGGTTCTAGGCACTCTGCAG  Reverse AGAGCCGATGTCAATCAGCAGG |
| *WNT7A* | Forward AGGAGAAGGCTCACAAATGGGC  Reverse CGGCAATGATGGCGTAGGTGAA |
| *EPB41* | Forward AGACCAGGCAAGCTAGTGCTCT  Reverse GTAATGGCAGGTGCAGAAGTGG |
| *CD40* | Forward CCTGTTTGCCATCCTCTTGGTG  Reverse AGCAGTGTTGGAGCCAGGAAGA |
| *FERMT1* | Forward TTGAAGATGGTGAGGTTGCGAGTC  Reverse GGGTTGGCTGAATGCGAGGATG |
| *hsa-miR-26a-5p (housekeeping)* | 477995_mir (Thermo Fisher Scientific) |
| *hsa-miR-429* | 477849_mir (Thermo Fisher Scientific) |
| *hsa-miR-200a-5p* | 478752_mir (Thermo Fisher Scientific) |
| *hsa-miR-200b-3p* | 477963_mir (Thermo Fisher Scientific) |
| *hsa-miR-149-5p* | 477917_mir (Thermo Fisher Scientific) |
| *hsa-miR-139-5p* | 478312_mir (Thermo Fisher Scientific) |
| *hsa-miR-9-5p* | 478214_mir (Thermo Fisher Scientific) |
| *hsa-miR-34a-5p* | 478048_mir (Thermo Fisher Scientific) |

**Supplementary table 4 Antibodies used for Immunoblotting**

| **Antibody** | **Order number** | **Application** |
| --- | --- | --- |
| p62 | sc-28359 (Santa Cruz) | WB |
| β-actin | A5441 (Sigma) | WB |
| Anti-Mouse IgG (H+L), AP Conjugate | s372b (Promega) | Secondary AB, WB |
