# Supplementary material for: p62 mRNA suppresses NLRP1 expression in cutaneous SCC cells through miR-34a-5p
Source: Cell Death Dis. 2025 Jul 1;16(1):465. doi: 10.1038/s41419-025-07785-9 (PMC12218052; doi:10.1038/s41419-025-07785-9)
Supplement: Supplementary file 3 — Legend for Supplementary Figure Fig. S1 [file 41419_2025_7785_MOESM3_ESM.docx]

**Supplementary Figure**

**Fig. S1 p62 mRNA regulates expression of miRNAs.**

RNA-seq data showing expression of miRNAs upon p62 protein or mRNA knockout.

p62.1 (protein only knockout), p62.2K (RNA knockout).
